# Bootstrapping of Corneal Optical Coherence Tomography Data to Investigate Conic Fit Robustness

**DOI:** 10.3390/jcm12103522

**Published:** 2023-05-17

**Authors:** Achim Langenbucher, Nóra Szentmáry, Alan Cayless, Lena Münninghoff, Adam Wylegala, Jascha Wendelstein, Peter Hoffmann

**Affiliations:** 1 Department of Experimental Ophthalmology, Saarland University, 66424 Homburg/Saar, Germany; 2 Dr. Rolf M. Schwiete Center for Limbal Stem Cell and Aniridia Research, Saarland University, 66424 Homburg/Saar, Germany; 3 Department of Ophthalmology, Semmelweis University, 1028 Budapest, Hungary; 4 School of Physical Sciences, The Open University, Milton Keynes MK 6AA, UK; 5 Augen- und Laserklinik Castrop-Rauxel, 44575 Castrop-Rauxel, Germany; 6 Pathophysiology Department, Medical University of Silesia, 40-752 Katowice, Poland; 7 Department of Ophthalmology, Johannes Kepler University, 4021 Linz, Austria

**Keywords:** cornea, model surface fit, anterior segment tomography, model parameter uncertainties, bootstrap techniques, robustness of surface fit, conoid surface, biconic surface

## Abstract

Background: Fitting of parametric model surfaces to corneal tomographic measurement data is required in order to extract characteristic surface parameters. The purpose of this study was to develop a method for evaluating the uncertainties in characteristic surface parameters using bootstrap techniques. Methods: We included 1684 measurements from a cataractous population performed with the tomographer Casia2. Both conoid and biconic surface models were fitted to the height data. The normalised fit error (height—reconstruction) was bootstrapped 100 times and added to the reconstructed height, extracting characteristic surface parameters (radii and asphericity for both cardinal meridians and axis of the flat meridian) for each bootstrap. The width of the 90% confidence interval of the 100 bootstraps was taken as uncertainty and quoted as a measure of the robustness of the surface fit. Results: As derived from bootstrapping, the mean uncertainty for the radii of curvature was 3 µm/7 µm for the conoid and 2.5 µm/3 µm for the biconic model for the corneal front/back surface, respectively. The corresponding uncertainties for the asphericity were 0.008/0.014 for the conoid and 0.001/0.001 for the biconic. The respective mean root mean squared fit error was systematically lower for the corneal front surface as compared to the back surface (1.4 µm/2.4 µm for the conoid and 1.4 µm/2.6 µm for the biconic). Conclusion: Bootstrapping techniques can be applied to extract uncertainties of characteristic model parameters and yield an estimate for robustness as an alternative to evaluating repeat measurements. Further studies are required to investigate whether bootstrap uncertainties accurately reproduce those from repeat measurement analysis.

## 1. Introduction

Corneal topographers have a long tradition in ophthalmology for measuring and visualising the corneal refractive power profile. The first such instruments (TMS-1, Computed Anatomy, New York, New York USA and EyeSys (Technomed, Germany) were launched in the early 1990s. A Placido pattern is projected to the cornea, reflected off the pre-corneal tear film and imaged by a camera. Measurement with Placido topographers requires an intact tear film, and this precludes eyes with insufficient tear film status. The first Scheimpflug tomographers were developed some years later. These project a sequence of rotating or scanning slits onto the cornea and capture the diffuse volume scattering in a similar way to a slitlamp biomicroscope. This measurement technique is capable of measuring the architecture of the entire anterior segment and is independent of the tear film status. The newest generation of corneal tomographers is based on optical coherence technology (OCT). Here, a low-coherence light source, mostly in the red or infrared region of the light spectrum, is projected onto the cornea, and the optical pathlength is compared to the pathlength of a reference beam in a setup similar to a Michelson interferometer.

With topographic/tomographic examinations, clinicians are mostly interested in the curvature data of the corneal front/front and back surface as well as in variations in the corneal power profile [1]. The principal characteristics of the corneal surface are typically described in terms of the corneal radius of curvature of both corneal surfaces in the flat and steep meridian, the orientation of the flat or steep meridian, and the asphericity [1,2,3,4,5,6]. From the curvature in both cardinal meridians and the orientation, we obtain the base curve or equivalent power and astigmatism with the axis of the torus. The overall or meridional asphericity gives advice, e.g., in contact lens fitting or in the selection of an appropriate lens design for cataract surgery [7,8,9].

In most cases, these characteristic data are derived from fitting a parametric model surface to the corneal topographic or tomographic data within a central region (e.g., in the 6 mm zone) [9,10,11]. However, we have to be aware that the eye (and, therefore, the cornea) is not necessarily aligned to the instrument axis of the measurement device. Therefore, in addition to the rotation of the surface around the Z axis, the tilt (around the X and Y axis) and decentration in X, Y, and Z have to be considered during the fit procedure [3,4,5,12]. Furthermore, we have to consider that topographic and tomographic data contain some noise, which may affect the characteristic surface parameters. However, in contrast to a focal measurement of corneal front surface curvature at four distinct points using a manual keratometer, extraction of the characteristic surface parameters may be based on thousands of data points, making the output more robust [13].

While there is no unique surface model for the data fit covering all the measurement devices on the market, all of them allow a direct data export in a common data file format, enabling users to program their own customised fit procedure. In general, fit surfaces are always a compromise between generating the most surface information (complex fit surfaces) and simplicity (simple fit surfaces with fewer parameters [7,8,9]). A surface fit with a complex surface provides detailed information about the surface but shows large variabilities and tends to overfit the surface [10,11]. In contrast, a simple surface fit provides only basic characteristics but is known to be very robust. As a rule of thumb, the fit surface should always be as simple as possible and as complex as necessary.

In the ideal situation where multiple measurements are available, the robustness of a corneal surface fit would normally be evaluated by performing repeat measurements on each patient and fitting the surface model to each set of data individually. The variation of the model parameters would then be used as a measure of the reliability (or robustness) of the model.

However, where measurements are made on individual patients in a clinical setting, it is not always practical to ask patients to undergo multiple repeat measurements in order to assess repeatability or variability. In this type of situation, bootstrapping is a frequently used technique for simulating and sampling the variation of model parameters using data from a population where repeat measurements are not feasible or practical. Bootstrapping involves building multiple samples from a single dataset of individual measurements by sampling with replacement (as opposed to subsampling) in order to simulate multiple samples taken from the population as a whole. 

The strategy used here follows this principle: after fitting an initial surface model, the fit error is evaluated and then resampled multiple times with replacement (i.e., bootstrapped). All of these fit errors are then superimposed onto the initial fit, generating a new set of data to which surface models are fitted. Statistical metrics such as median or confidence intervals of the fit parameters are extracted from these bootstrapped models, and analysed as an estimate of the robustness of the model. 

If our goal is to extract curvatures in two orthogonal meridians, together with the orientation of these meridians and the asphericity, then a simple conic model surface seems to be sufficient [6,7,8,9]. This surface covers all 2nd order surfaces, such as ellipsoids, hyperboloids, and paraboloids, with a strict coupling between the asphericity in both meridians. Fortunately, there is a straightforward method to fit a conoid, including decentration and rotations with respect to the X, Y, and Z coordinates. By contrast, using a biconic as the model surface allows the extraction of the asphericity in both meridians independently. This is, however, a bit more complex [9,10,11] as there is no straightforward (algebraic) technique for fitting a biconic surface, including all six degrees [5,12] of freedom (decentration and rotations) to measurement data.

The purpose of this study was as follows:

- To present a method for extracting corneal front and back surface measurement data from an OCT-based corneal tomographer and to fit a simple conoid surface model to obtain the curvature in both cardinal meridians together with the orientation and the overall asphericity of the conoid and the alignment of the conoid in terms of apex decentration in X, Y, Z, and tilt (rotation around X and Y);
- To use the alignment data from the previous step (apex decentration in X, Y, Z, and tilt (around X and Y)) and rotation (around Z of the conic) to fit a biconic model surface to the tomographic measurement data in order to obtain the curvature and the asphericity separately for both cardinal meridians;
- To evaluate the robustness of the conic and biconic surface model fit in terms of 90% confidence intervals (apex decentration, tilt, rotation, curvatures, and asphericities) using bootstrapping techniques;
- To apply this analysis to a large dataset of anterior segment OCT data extracted from the Casia2 with measurements from a cataractous population.

## 2. Materials and Methods

### 2.1. Dataset for Our Data Analysis

In this retrospective study, a data download containing 5636 measurements from the Augen- und Laserklinik Castrop-Rauxel, Castrop-Rauxel, Germany, was assessed. The local ethics committee (Ärztekammer des Saarlandes) provided a waiver for this study (157/21). The data were filtered at the source for measurements prior to cataract surgery. Duplicate measurements of eyes were discarded from the dataset. Where measurements of both eyes were available, one eye was selected randomly for consideration in our evaluation. After filtering, the raw export data (.CSV-format) containing 1844 measurements were transferred to us in an anonymised fashion, precluding back-tracing of the patient. The anonymised data contained tomographic measurements acquired using the Casia2 (Tomey GmbH, Nürnberg, Germany, software version Ver.50.5A.03). The CSV data were imported into MATLAB (Matlab 2021, MathWorks, Natick, MA, USA) for further processing.

### 2.2. Preprocessing of the Data

Custom software was written in Matlab. In the standard export application of the Casia2 software, the data included lateral position data and data on axial, keratometric, or instantaneous curvature/power of both surfaces or real/refractive power of the cornea, as well as height and elevation data. In addition to eye side (OS or OD), gender, and age, the data selection was restricted to the lateral position and height of the corneal front and back surface, and all other data were discarded. Each data block (radial position r and surface height (Z) of both surfaces) contained cylinder coordinate measurements at 32 semi-meridians (angle θ from 0° to 348.75° in steps of 11.25°) with 400 radial positions (radial distance r from centre from 0.02 to 8.0 mm in steps of 0.02 mm) each in a central 16 mm zone. Measurements with a quality marker QS other than ‘OK’ [13] or incomplete datasets for the corneal front or back surface height within the 7 mm central zone of the cornea were excluded from the study.

### 2.3. Surface Fit with a Conoid and a Biconic to the Corneal Front and Back Surface

The strategy of fitting model surfaces to the measurement data of the corneal front and back surfaces was identical. Cylindrical coordinates (r,θ,Z) restricted to the central 6 mm zone (150 × 32 data points both for the front and back surface) were converted to Cartesian coordinates (X,Y,Z) for further processing [7,8]. With a formulation of a conoid as a quadric surface

(1) $$\left(\begin{array}{ccc} X & Y & Z \end{array}\right)\cdot \left[\begin{array}{ccc} a_{11} & \frac{a_{12}}{2} & \frac{a_{13}}{2}\\ \frac{a_{12}}{2} & a_{22} & \frac{a_{23}}{2}\\ \frac{a_{13}}{2} & \frac{a_{23}}{2} & a_{33} \end{array}\right]\cdot \left(\begin{array}{c} X\\ Y\\ Z \end{array}\right)+\left(\begin{array}{ccc} b_{1} & b_{2} & b_{3} \end{array}\right)\cdot \left(\begin{array}{c} X\\ Y\\ Z \end{array}\right)+c=0$$

or

(2) $$a_{11}\cdot X^{2}+a_{22}\cdot Y^{2}+a_{33}\cdot Z^{2}+a_{12}\cdot XY+a_{31}\cdot XZ+a_{23}\cdot YZ+b_{1}\cdot X+b_{2}\cdot Y+b_{3}\cdot Z+c=0$$

where *a*_11_…*a*_33_ refer to the elements of the 3 × 3 matrix A and b_1_…b_3_ to the elements of the vector b and c to a constant (intercept) [10,11]. The elements of matrix A and vectors b and c can be directly extracted from the measurement data X, Y, and Z (with data points x_1_…x_M_, y_1_…y_M_, and z_1_…z_M_). e.g., solving the following linear equation system [10,11]:


(3) $$\left[\begin{array}{ccccccccc} x_{1}^{2} & y_{1}^{2} & x_{1}y_{1} & x_{1}z_{1} & y_{1}z_{1} & x_{1} & y_{1} & z_{1} & 1\\ \ldots & \ldots & \ldots & \ldots & \ldots & \ldots & \ldots & \ldots & \ldots \\ x_{M}^{2} & y_{M}^{2} & x_{M}y_{M} & x_{M}z_{M} & y_{M}z_{M} & x_{M} & y_{M} & z_{M} & 1 \end{array}\right]\cdot \left[\begin{array}{c} \begin{array}{c} a_{11}\\ a_{22}\\ a_{12} \end{array}\\ \begin{array}{c} a_{13}\\ a_{23}\\ b_{1} \end{array}\\ \begin{array}{c} b_{2}\\ b_{3}\\ c \end{array} \end{array}\right]=\left[\begin{array}{c} -z_{1}^{2}\\ \ldots \\ -z_{M}^{2} \end{array}\right]$$

Assuming the conoid in its canonical form with semi-axes sa_1_, sa_2_, and sa_3_ and translated and rotated coordinates X_c_, Y_c_, and Z_c_

(4) $$\frac{X_{c}^{2}}{sa_{1}^{2}}+\frac{Y_{c}^{2}}{sa_{2}^{2}}+\frac{Z_{c}^{2}}{sa_{3}^{2}}=1$$

The conversion of coordinates from measurement coordinate system X/Y/Z to X_c_/Y_c_/Z_c_ and vice versa is described by a rotation matrix U and a translation vector T with:

(5) $$\begin{array}{ccc} X & = & U\cdot \left(X_{c}+T\right)\\ X_{c} & = & U^{-1}\cdot X-T=U^{\prime }\cdot X-T \end{array}$$

In this context, (.)′ refers to the transpose of (.), and matrix A is identified as a product of A = U′·L·U with the rotation matrix U and the 3 × 3 diagonal matrix L. Rotation matrices (like U) are known to be positive Hermitian with a determinant of 1 and the inverse of the matrix (.)^−1^ equals the transpose (.)′. With eigenvalue decomposition, U refers to the 3 × 3 matrix with the eigenvectors, and L to the 3 × 3 diagonal matrix with the eigenvalues. 

From the parameter vector [a_11_ a_22_ 1 a_12_ a_13_ a_23_ b_1_ b_2_ b_3_ c]′ derived from Equation (3) and completed by element 3, matrix A as defined in Equation (1) is evaluated with an eigenvalue decomposition, and the 3 Euler angles α (tilt of the conoid around X), β (tilt of the conoid around Y), and γ (rotation of the conoid around Z, orientation of the principal meridian or astigmatism axis) were extracted [6,10,11]. The eigenvectors (the 3 elements of the diagonal matrix L) define the semi-axes of the ellipsoid in the canonical form sa_1_, sa_2_, and sa_3_. From the location of the centre of the ellipsoid (T) and the semi-axes sa_1_, sa_2_, and sa_3_ the location of the apex of the conoid (with coordinates CA_x_, CA_y_, and CA_z_) is derived, and with sa_1_, sa_2_, and sa_3_ and the constant c the radius of curvature in both principal meridians (Rx and Ry) and the asphericities (Qx and Qy) are calculated.

(6) $$\begin{array}{c} \begin{array}{ccc} CR_{x} & = & \frac{sa_{1}^{2}}{sa_{3}}\\ CR_{y} & = & \frac{sa_{2}^{2}}{sa_{3}} \end{array}\\ \begin{array}{ccc} CQ_{x} & = & \frac{sa_{1}^{2}}{sa_{3}^{2}}-1\\ CQ_{y} & = & \frac{sa_{2}^{2}}{sa_{3}^{2}}-1 \end{array} \end{array}$$

As can be seen from Equation (6), the asphericities CQx and CQy are coupled and cannot be calculated independently [9,10,11]. The coordinates X/Y/Z are then transformed to the coordinate system of the canonical form of the conoid X_c_/Y_c_/Z_c_ using Equation (5), and the fit error E_C_ for the conoid derived as the difference between Z_c_ and the reconstructed conoid ZCR_C_ surface in canonical form:

(7) $$\begin{array}{ccc} ZCR_{C} & = & sa_{3}\cdot \sqrt{1-\frac{X_{c}^{2}}{sa_{1}^{2}}+\frac{Y_{c}^{2}}{sa_{2}^{2}}}\\ E_{C} & = & Z_{c}-ZCR_{C} \end{array}$$

In the next step, the coordinate data corresponding to the canonical form of the conoid X_c_/Y_c_/Z_c_ were used to fit a parametric biconic surface model [9] aligned to the orientation of the conoid. In a general form, a parametric biconic surface with a height Z_B_ is defined by:

(8) $$Z_{B}=Bz_{0}+\frac{\frac{X_{c}^{2}}{BR_{x}}+\frac{Y_{c}^{2}}{BR_{y}}}{1+\sqrt{1-\left(1+BQ_{x}\right)\frac{X_{c}^{2}}{BR_{x}{}^{2}}-\left(1+BQ_{y}\right)\frac{Y_{c}^{2}}{BR_{y}{}^{2}}}}$$

The biconic defined in Equation (8) was fitted to the data X_c_/Y_c_/Z_c_ using nonlinear iterative optimisation. The 5 parameters of the biconic (offset of the apex of the biconic to the apex of the conoid Bz_0_, both radii of curvature BR_x_ and BR_y_ and asphericities BQ_x_ and BQ_y_ [9]) were determined using the Levenberg-Marquardt algorithm [14,15] by minimising the sum of squared errors $\underset{1\ldots M}{\sum }{\left(Z_{c}-Z_{B}\right)}^{2}$. The reconstructed biconic surface in the canonical coordinate system of the conoid ZBR_C_ is defined by ZBR_C_ = Z_B_ by inserting the parameters (BR_x_, BR_y_ BQ_x_, and BQ_y_) derived from the Levenberg–Marquardt optimization [14,15] into Equation (8), and the fit error E_B_ is derived from the difference between the Z_C_ and the reconstructed surface height:

(9) $$E_{B}=Z_{C}-ZBR_{C}=Z_{C}-Bz_{0}-\frac{\frac{X_{c}^{2}}{BR_{x}}+\frac{Y_{c}^{2}}{BR_{y}}}{1+\sqrt{1-\left(1+BQ_{x}\right)\frac{X_{c}^{2}}{BR_{x}{}^{2}}-\left(1+BQ_{y}\right)\frac{Y_{c}^{2}}{BR_{y}{}^{2}}}}$$

### 2.4. Implementation of Bootstrapping for Robustness Evaluation

Since the anterior segment OCT measurement data include noise, a bootstrapping strategy [16,17] was implemented to evaluate the effect of measurement noise on the extracted parameters of both surface models and to evaluate the robustness of the conoid and biconic fits. Bootstrapping was implemented separately for the conoid and the biconic fits for both the corneal front and back surface, with measurement data transformed to the canonical coordinate system [10,11] defined by the orientation of the conoid (X_c_/Y_c_/Z_c_). For both surface models and surfaces, a sequence of N_B_ = 100 bootstraps was used to assess the uncertainties of the fit parameters [18,19,20]. Our bootstrap process (shown here for the conoid example) included the following steps:

(1) The fit error E_C_ (Equation (7)) or E_B_ (Equation (9)), which was evaluated in a pilot study to increase over the distance from the centre r (E_C_ ~ d_0_ + d_2_·r²; d_0_ = 0.04 and d_2_ = 1.05; r² = x² + y²), was normalised to E_C0_ = E_C_/(d_0_ + d_2_·r²) and E_B0_ = E_B_/(d_0_ + d_2_·r²) to omit heteroscedasticity of the fit error over r [10,11].
(2) The fit error E_C0_ or E_B0_ was sampled N_B_ times with replacement (E_C0_1 to E_C0_N_B_ or E_B0_1 to E_B0_N_B_).
(3) The normalisation [11] of the N_B_ bootstrapped fit errors E_C0_1 to E_C0_N_B_ (or E_B0_1 to E_B0_N_B_) was reversed (shown here for the conoid example) to obtain E_C0_1′ … E_C0_N_B_′

(10) $$\begin{array}{ccc} E_{C0}1\prime & = & E_{C0}1\cdot \left(d_{0}+d_{2}\cdot r²\right)\\ \ldots & \ldots & \ldots \\ E_{C0}N_{B}\prime & = & E_{C0}N_{B}\cdot \left(d_{0}+d_{2}\cdot r²\right) \end{array}$$

(4) The bootstrap errors after reversion E_C0_1′ to E_C0_N_B_′ (or E_B0_1′ to E_B0_N_B_′) were added to the conoid or biconic surface reconstructions ZCR_C_ or ZBR_C_. For each bootstrap, a new conoid or biconic surface fit was performed to generate the characteristic surface parameters. In total, N_B_×2×2 = 400 surface fit cycles were processed for each examination for 100 bootstraps, 2 surfaces, and 2 surface models.
(5) The 90% confidence intervals [18,19,20] were derived from the N_B_ sets of surface fit parameters for the conoid (CR_x_, CR_y_ CQ_x_, CQ_y_, and γ) and the biconic (BR_x_, BR_y_ BQ_x_, and BQ_y_). For the conoid rotation angle γ, which shows periodicity at 180°, a cyclic correction was applied to evaluate the robustness of the astigmatic axis. The 90% confidence interval was quoted in this context as the ‘uncertainty’ of the surface parameters [18].

### 2.5. Postprocessing of Data and Statistical Evaluations

For each of the surface representations (conoid and biconic) the refractive power of the corneal front surface in both cardinal meridians CP_x_, CP_y_, BP_x_, and BP_y_ was calculated based on the curvature radii CR_x_, CR_y_, BR_x_, and BR_y_ and using *n* = 1.376 as the refractive index (Liou-Brennan schematic model eye [21]). Accordingly, the power of the corneal back surface was calculated using refractive indices of aqueous humour n = 1.336 and cornea n = 1.376. In addition, the mean power, the astigmatic power, and the vector components projected to the 0/90° and the 45/135° meridian were derived for both corneal surfaces from CP_x_, CP_y_, BP_x_, BP_y_, and the rotation angle γ indicating the orientation of the conic surface fit [6,7,8,9]. For both corneal surfaces, explorative data are shown with mean, standard deviation (SD), median, and 90% confidence intervals (5% quantile as the lower bound and 95% quantile as the upper bound) for the conoid and the biconic surface fit to the corneal front and back surface. The fit errors for the conoid (E_C_) and the biconic (E_B_) for both corneal surfaces are described by mean, SD, median, and root mean squared value (rms). The width of the 90% confidence interval [18,19,20] of the N_B_ bootstraps as a measure of the robustness of the fit to both corneal surfaces was evaluated for both the conoid (surface parameters CR_x_, CR_y_ CQ_x_, CQ_y_, and γ) and the biconic (surface parameters BR_x_, BR_y_ BQ_x_, and BQ_y_) models.

## 3. Results

From the N = 1844 data from the Casia2 tomographer transferred to us, a total of N = 1684 (911 right eyes and 773 left eyes) were used after eliminating measurements with incomplete data or with a quality check other than ‘OK’. The mean age at the time of measurement was 70.63 ± 12.88 years.

**Figure 1:**
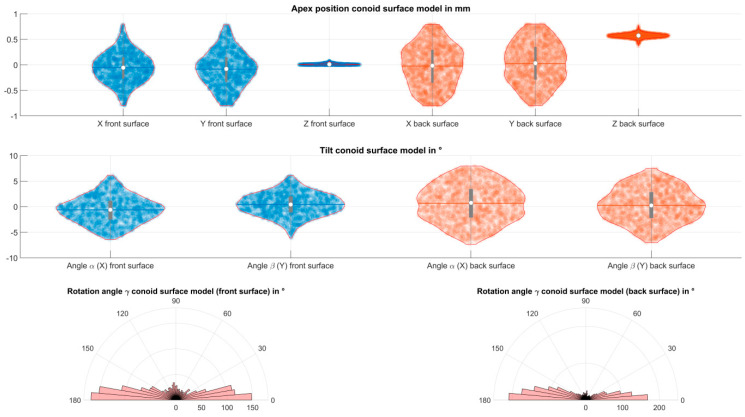
Alignment of the conoid surface fit to the corneal front surface (**left** side) and back surface (**right** side) measurement data. The position of the apex with respect to the coordinate system of Casia2 is shown in the upper plot. The tilt of the conoid (rotations around the X and Y axis) is shown in the middle plot. The violin plots display the distribution together with the data spread, the mean, the median, and the 90% confidence interval. The polar histograms in the lower plot show the angular distribution of the flat axis of the conoid (rotation around the Z axis, astigmatism axis).

Table 1 shows the position of the apex in the coordinate system of the Casia2 (X/Y/Z) for the conoid and biconic fits to the corneal front and back surface data. The X and Y components refer to lateral decentration of the surface, whereas the Z component refers to the axial displacement. The central corneal thickness can be extracted directly from the difference between the axial displacements of the corneal front and back surface fits. The axis alignment of the conoid, as shown with the tilt components (rotations around the X and Y axis), was used to transform the coordinates of the Casia2 to the coordinates of the canonical form (X_C_/Y_C_/Z_C_). Figure 1 displays the data spread and distribution of the apex position for both surface fit models and corneal surfaces together with the mean, median, and 90% confidence intervals in a violin plot in the upper graph. The tilts of the conoid around the X and Y axis in the middle plot indicate that the conoids are typically somewhat skewed relative to the coordinate system of Casia2. From the lower plot, we can see that the flat axis of the conoid (the cylinder axis γ) around the horizontal meridian (0°/180°, astigmatism with the rule) is much more frequent in the dataset compared to oblique axis (45°/135° meridian) or to an astigmatism against-the-rule (90°/270° meridian).

**Figure 2:**
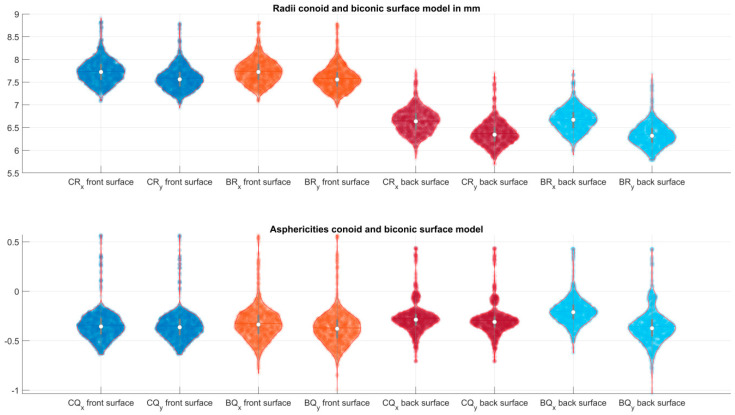
Violin plots displaying the distributions, the mean, median, and the 90% confidence intervals of the radii of curvature and the asphericities for the flat and steep meridian for the conoid and the biconic surface fit to the corneal front (**left** side) and back surface (**right** side) data. In the canonical form, X refers to the flat meridian, Y to the steep meridian. In the upper plot, the radii of curvature are shown, and in the lower plot, the asphericities.

Table 2 lists the explorative data for the radii of curvature and the asphericity in the flat (X) and the steep (Y) meridian for a conoid and biconic surface fitted to the corneal front and back surface data of the Casia2. With the conoid fit, both asphericities are linked together due to the definition of the quadric surface, whereas for the biconic fit, the radii of curvature and asphericities in both principal meridians were fitted independently. Figure 2 displays the data scatter and the distributions of the radii of curvature and the asphericity in the flat (X) and steep (Y) meridian for the conoid and biconic model surface fitted to the measurement data. We can see directly from the plot that the radii of curvature at the corneal front surface are much larger as compared to the radii of curvature at the corneal back surface with both surface models but that there is no systematic difference between the asphericities for the corneal front and back surface or between the flat and steep meridian of both surface models.

**Figure 3:**
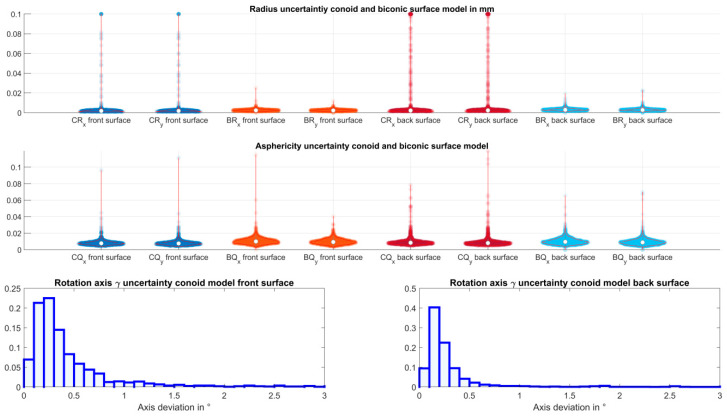
Uncertainty (width of the 90% confidence interval) as a measure for the robustness of the fit of the corneal front and back surface Casia2 measurement data with a conoid and biconic surface model. The confidence interval was derived from bootstrapping the data with N_B_ = 100 sequences. The upper graph shows the violin plot with the data scatter, the distribution, and the mean, median, and confidence interval for the flat (X) and steep (Y) meridians from the bootstrapped radii of curvature, the middle plot the respective graph for the asphericities. The lower graph displays the histograms of the bootstrapped variation in the flat axis of the conoid for the front (**left** side) and back surface (**right** side). The 180°periodicity of the conoid axis was considered in the plot.

Table 3 gives an overview of the explorative data for the uncertainties of the surface parameters using bootstrapping. Using a sequence of 100 bootstraps of the fit error, the variation of both surface models fitted to the corneal front and back surface data was evaluated as the width of the 90% confidence interval. We can see from the Table that the uncertainty of the radii of curvature extracted from the conoid fit shows a mean variation of around 3 µm/7 µm for the corneal front/back surface and, correspondingly, around 2.5 µm/3 µm for the radii of curvature extracted from the biconic fit. However, if the 95% quantile is considered, the uncertainty in the radii extracted from the conoid increases to around 50 µm for the front surface fit and 1/10 of an mm for the back surface fit. For the radii extracted from the biconic surface fit, the respective values for the corneal front/back surface increase to around 40 µm/50 µm. Figure 3 shows the data spread and distributions of the uncertainties of the bootstrapped radii of curvature (upper graph) and asphericities (middle graph) for the conoid and the biconic surface model fitted to the corneal front and back surface data. From the distributions, we find that there might be some extreme values and outliers where the surface fit with a conoid or biconic is not really stable. The lower graph shows the uncertainty of the orientation of the flat axis of the conoid. From this graph, we can see that the axis can be extracted from the conoid model with high reproducibility, with the variation between the bootstraps in a range of up to 3°.

**Figure 4:**
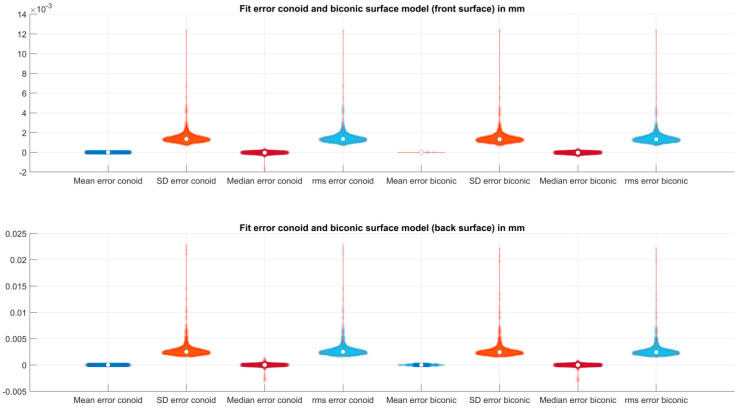
Violin plot showing the data spread, distribution, mean, median, and 90% confidence intervals of the mean, standard deviation (SD), median, and root mean squared fit error for the corneal front surface (**upper** plot) and the corneal back surface (**lower** plot). The fit error for the conoid surface fit/biconic surface fit is displayed on the left side/right side.

Table 4 lists the explorative data for the fit error of the conoid and the biconic surface model to both corneal surfaces. There is no systematic offset of the fit error (the mean and median error of the conoid and biconic fit are around 0). However, the most important parameters are the SD and rms fit errors, which are, on average, around 1.4 µm/2.3 µm for the corneal front/back surface with the conoid surface model and around 1.4 µm/2.2 µm for the biconic surface model. Considering the 95% quantile, the mean SD fit error increases to 2.3 µm/4.8 µm for the conoid surface model and to 2.2 µm/4.5 µm for the biconic surface model fitted to the corneal front/back surface. In Figure 4, we see from the distributions of the mean, SD, median, and rms fit error that there is no systematic offset, but also that in some (rare) cases, the SD or rms fit error is much higher compared to the ‘normal’ fit error of around 1–2 µm. 

**Figure 5:**
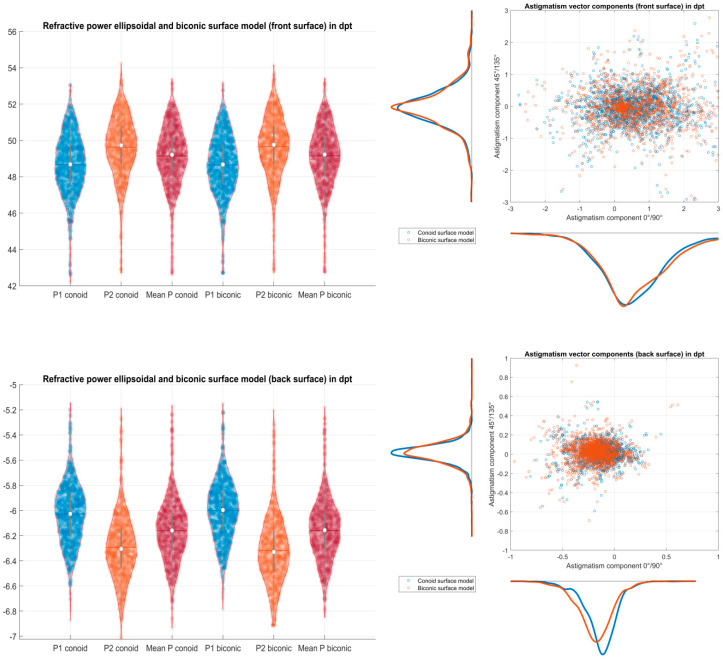
Refractive power derived from the radius of curvature data from the conoid and biconic fits the corneal front and back surface. Based on the refractive indices for air, cornea, and aqueous humour from the Liou–Brennan schematic model eye, the power of the corneal front surface (**upper left** graph) and the corneal back surface (**lower left** graph) is shown for the flat meridian (X), the steep meridian (Y), and the mean power with violin plots. The decomposition of astigmatism into vector components (**double angle** plot) is displayed for both surface models for the corneal front surface (**upper right** graph) and the corneal back surface (**lower right** graph) with scatterhistograms.

Figure 5 displays on the left graph the corneal power data extracted from the radii data of both corneal surface models fitted to the corneal front and back surfaces. Based on the refractive indices derived from a schematic model eye, the refractive power of the corneal front/back surface ranges around 49 dpt/−6 dpt. On average, there is only a small difference between the refractive power of the flat (X) and the steep meridian (Y). Decomposing astigmatism as the difference between the refractive powers of the steep and the flat meridians into vector components, the double angle plots in the right graph indicate that the distribution for astigmatism at the corneal front surface is systematically shifted to the right, and the distribution for astigmatism at the corneal back surface is shifted to the left. This indicates that at the corneal front surface, we frequently have an astigmatism with the rule, whereas, at the corneal back surface, we mostly have astigmatism against the rule.

## 4. Discussion

Corneal tomographers are well established in clinical routine for analysing the entire anterior segment of the eye with a focus on both corneal surfaces. Especially for the diagnosis of corneal pathologies, these tomographic measurements assist classical slit lamp examination, and many indices and markers are derived from the devices. In contrast to simulated keratometry values (SimK [13]), which aim to replicate the measures of a manual keratometer by providing the local corneal power at four distinct locations in the mid periphery (two points each at two cardinal meridians), the tomographer is capable of extracting corneal curvature from thousands of data points which should, in general, be much more robust compared to SimK [13]. In addition, as the entire corneal front and the back surface are measured, the asphericity as an increase or decay of refractive power from the centre to the periphery can easily be extracted from the data [3,4]. For that purpose, a model surface has to be fitted to the measured data points. This parametric model surface should be individually adapted to the requirements, meaning that if we were only interested in the average radius of curvature of the corneal surface, a simple floating sphere as the model surface would be sufficient, and tilts or rotations would not have to be considered. However, in most clinical applications, such a simple model surface is not sufficient for the characterisation of the cornea [3,4,6,7,8,9]. If we wish to extract the average central curvature together with the average asphericity, we require at least a rotational symmetric conoid (two-axis conoid), and for the surface fit to the data points, we have to consider both displacements of the model surface and tilt (rotations around X and Y). However, in most clinical applications, we cannot restrict the models to rotationally symmetric surfaces as we are also interested in surface astigmatism [6]. The simplest model surface for those applications is a three-axis conoid (quadric surface), which provides the central radius of curvature in two cardinal meridians together with the axis of the flat (or steep) meridian and the asphericity [22]. However, we have to be aware that the canonical form of a conoid is restricted to three degrees of freedom, meaning that if the radii of curvature are selected independently, we have only one degree of freedom left for the asphericity, and therefore the asphericity in both meridians cannot be selected independently. In a more general case, if we are interested in extracting the radii of curvature and the asphericity independently for both cardinal meridians [9], we have to use a biconic surface model. However, in this case, we have to be aware that the surface fit with a biconic model is more complex as we are no longer dealing with a quadratic function, which leads to a solution of a least squares matrix problem [9]. We also have to be aware that the more individual the surface, the less robust is our surface fit. If we would like to go into even more detail and extract additional characteristic surface data, we could add some higher order polynomial terms [2,10,22] to the biconic model surface (even order radial symmetric polynomials starting with radial order 4) or add Zernike expansion terms to consider individual fluctuations of the corneal refractive power profile.

However, we have to keep in mind that, in reality, the corneal surfaces are not fully represented by such a model surface, and in addition, all measurement data of the corneal surface are somewhat contaminated with noise. For optical measurement, the uncertainty of the data points (as a difference in height Z) typically increases from centre to periphery [11] as we deal with a convex surface for two reasons: (1) if we assume that the uncertainty of the data points perpendicular to the surface is constant over the surface, the uncertainty in Z increases from centre to periphery, and (2) the back-reflection of light is strongest in the centre which means that the signal to be evaluated in the measurement is much weaker in the periphery [13]. Both the variations of the corneal shape from the ‘perfect’ surface model and the measurement noise affect the precision of the surface fit (parameters). For this purpose, we implemented a bootstrapping strategy that provides an estimate for the uncertainty of the surface model parameters with ‘imperfect’ data and measurement noise [18,19,20]. The surface measurement data are sampled N_B_ times with replacement, meaning that in each of the N_B_ data sequences, a subset of the measurement data (with duplicates) is considered. Then, for each of the N_B_ sequences, a surface fit is implemented, and the characteristic surface parameters are extracted. The width of the confidence intervals of the characteristic surface parameters (e.g., radii of curvature in both meridians) as a measure for the uncertainty is used as a metric for the robustness of the fit. In this study, we bootstrapped the fit error of the initial surface characterisation N_B_ = 100 times. The bootstrapped fit error was added to the model surface reconstruction [18] of the initial surface fit, and those data were used to retrieve the parameters of the model surface. Finally, the 90% confidence interval [18,20] of the N_B_ = 100 characteristic surface parameter sets was quoted as the uncertainty of the fit. In this context, we have to consider that the fit error increases from the centre to the periphery, which makes normalisation of the fit error before bootstrapping necessary. In a pilot study, we evaluated the typical behaviour of the fit error in the radial direction and found that for both corneal surfaces, the fit error in the radial direction is represented by a simple polynomial of 2nd order with an intercept d_0_ and a quadratic term d_2_. This polynomial was used for normalisation before bootstrapping the fit error, and the normalisation was reversed before adding the bootstrapped fit error to the reconstructed surface data of the initial fit [11].

In the current study, we used a large dataset from the Casia2 anterior segment tomographer. The dataset included measurements of elderly patients before cataract surgery (only one eye measured per patient), and duplicate measurements per eye were filtered out. All data were checked for the proper quality marker provided by the Casia2 software as well as for complete data in the 7 mm zone. Finally, we restricted our analysis to the data within the central 6 mm zone. The zone to be considered in such data analysis is always a compromise between the robustness of the surface fit (the larger, the more robust) and the relevance of the data for the visual performance (central and paracentral data over the entrance pupil are most relevant for vision). The measurement data were first used to fit a conoid surface [6]. From this conoid surface, we extracted the radii of curvature and the asphericity in both cardinal meridians, the decentration of the centre, the location of the apex, and the orientation in terms of tilt angles around X and Y (α and β) and the rotation around Z (angle γ) as the orientation of the flat meridian [10,11]. The apex location and orientation angles of the conoid were used to transform the coordinates of the measurement data to the canonical form (X_C_/Y_C_/Z_C_). This representation of the measurement data was used to extract the surface parameters of the biconic surface as a general calculation strategy for extracting a biconic with four surface parameters (BR_x_, BR_y_, BQ_x_, BQ_y_) together with six degrees of freedom (displacement of the apex in X/Y/Z and orientation angles α/β/γ) proved to yield unstable results in some cases.

After fitting initial parametric conoid and biconic surface models to the corneal front and back surface measurement data, bootstrapping [16,17] was used for both surface models and corneal surfaces to investigate the uncertainty of the surface fit parameters. We found that the mean uncertainty of the radii of curvature for the conoid surface model was in the range of 3 µm for the front surface and 7 µm for the back surface. For the biconic surface model, the uncertainty was slightly lower in a range of 2.5 for the front surface and 3 µm for the back surface. As the biconic fit has four instead of three degrees of freedom in the canonical form of the surface, it is obvious that the uncertainties are equal or lower compared to the conoid surface fit. We also found that the mean uncertainty of the asphericity for the conoid surface model was in the range of 0.008 for the front surface and 0.015 for the back surface. For the biconic surface model, the uncertainty was in the range of 0.01 for the corneal front and back surfaces. Referenced to the absolute value of corneal asphericity, which is around −0.22 according to the Liou–Brennan schematic model eye [21], the relative uncertainty is much higher compared to the relative uncertainty of the radii. However, we learn from the results that the uncertainty of the extracted radii and asphericities of both surface models for both corneal surfaces for a ‘normal elderly population’ measured before cataract surgery is quite below any clinical significance. With some corneal pathologies, such as keratoconus, the uncertainty might be much larger. From our data, we also see that the mean rms fit error as the root mean squared height difference between the measured data and the reconstructed data in the canonical coordinates is around 1.4 µm/2.7 µm for the conoid surface model and 1.4 µm /2.3 µm for the biconic surface model corneal front/back surface. We feel that this fit error is quite low, indicating that we included only ‘normal cases’ without corneal pathologies in our study. However, this also makes it clear that the fit error is systematically larger for the corneal back surface as compared to the corneal front surface, which might be due to the fact that the back surface is always imaged through the front surface as a refracting element and irregularities at the corneal front surface may, therefore, affect the back surface measurement.

In conclusion, this study shows a strategy for fitting a conoid or biconic model surface to the anterior segment OCT measurement data of the corneal front and back surface and for assessing the robustness of the surface fit for both surface models and the corneal front and back surface. Instead of using standard techniques based on evaluating a sequence of repeat measurements, we extracted the uncertainties of the characteristic surface parameters such as radii of curvature and asphericity in both cardinal meridians and the orientation of the flat meridian (astigmatic axis) as metrics for robustness by bootstrapping the fit error. However, comparative studies in the future would be needed to validate whether bootstrapping of the fit error of one single examination yields equivalent results for uncertainties of characteristic surface parameters or surface fit robustness as compared to the evaluation of repeat measurements.

## Figures and Tables

**Table 1:** Explorative data of apex position (conoid fit and biconic fit) in the X/Y/Z coordinates and the tilt of the conoid surface fit around the X and Y axis. As the alignment of the conoid surface (coordinates X_C_/Y_C_/Z_C_) was used to fit the biconic surface, the tilt angles α and β are identical. SD refers to the standard deviation and Quantile 5% and Quantile 95% to the lower and upper bound of the 90% confidence interval.

Apex Position and Tilt N = 1684		Apex Position Conoid Surface Fit			Apex Position Biconic Surface Fit			Tilt Conoid Surface Fit	
		X in mm	Y in mm	Z in mm	X in mm	Y in mm	Z in mm	X (α) in °	Y (β) in °
Corneal front surface	Mean	−0.0538	−0.0834	0.0142	−0.0538	−0.0834	0.0142	−0.6274	0.4045
	SD	0.2976	0.3460	0.0131	0.2976	0.3460	0.0131	2.5971	2.2845
	Median	−0.0557	−0.0795	0.0098	−0.0557	−0.0795	0.0097	−0.6021	0.4263
	Quantile 5%	−0.6483	−0.7366	0.0004	−0.6483	−0.7366	0.0004	−5.4580	−4.0041
	Quantile 95%	0.5569	0.6313	0.0474	0.5569	0.6313	0.0474	4.6602	4.7682
Corneal back surface	Mean	−0.0242	0.0246	0.5723	−0.0242	0.0246	0.5723	0.6568	0.2795
	SD	0.4023	0.4013	0.0440	0.4023	0.4013	0.0440	3.5610	3.2290
	Median	−0.0169	0.0318	0.5724	−0.0169	0.0318	0.5724	0.7532	0.2397
	Quantile 5%	−0.7447	0.7091	0.4844	−0.7447	−0.7091	0.4844	−5.7933	−5.8114
	Quantile 95%	0.7204	0.7302	0.6587	0.7214	0.7302	0.6589	7.0155	6.0655

**Table 2:** Explorative data of radii of curvature in both principal meridians (flat meridian in X and steep meridian in Y) derived from the canonical form of the conoid surface (left columns) and the biconic surface (right columns) for the corneal front and back surface. SD refers to the standard deviation and Quantile 5% and Quantile 95% to the lower and upper bound of the 90% confidence interval.

Radii of Curvature and Asphericities N = 1684		Radii of the Conoid Surface Fit		Asphericity of the Conoid Surface Fit		Radii of the Biconic Surface Fit		Asphericity of the Biconic Surface Fit	
		CR_x_ in mm	CR_y_ in mm	CQ_x_	CQ_y_	BR_x_ in mm	BR_y_ in mm	BQ_x_	BQ_y_
Corneal front surface	Mean	7.7350	7.5852	−0.3440	−0.3511	7.7384	7.5799	−0.3265	−0.3706
	SD	0.2696	0.2610	0.1588	0.1561	0.2689	0.2614	0.1703	0.1730
	Median	7.7235	7.5609	−0.3564	−0.3637	7.7243	7.5559	−0.3366	−0.3784
	Quantile 5%	7.2959	7.1816	−0.5827	−0.5846	7.3001	7.1679	−0.6050	−0.6441
	Quantile 95%	8.3314	8.1642	0.1109	0.0986	8.3480	8.1377	0.1198	0.0913
Corneal back surface	Mean	6.6458	6.3670	−0.2723	−0.2940	6.6774	6.3422	−0.2002	−0.3559
	SD	0.2697	0.2670	0.1536	0.1512	0.2643	0.2694	0.1445	0.1831
	Median	6.6375	6.3439	−0.2873	−0.3099	6.6707	6.3196	−0.2115	−0.3737
	Quantile 5%	6.1832	5.9262	−0.5290	−0.5376	6.2035	5.8910	−0.4419	−0.6690
	Quantile 95%	7.2433	7.0296	0.1323	0.0846	7.2622	7.0139	0.2022	0.0813

**Table 3:** Explorative data of the width of the 90% confidence intervals of the bootstrap samples (uncertainties) as a measure for the robustness of the surface fit. The confidence intervals for the radii and asphericity in the flat (in X) and steep meridian (in Y) were derived from N_B_ = 100 bootstraps from the canonical form of the conoid surface (left columns) and the biconic surface (right columns) for the corneal front and back surface. SD refers to the standard deviation and Quantile 5% and Quantile 95% to the lower and upper bound of the 90% confidence interval.

Radii and Asphericity Uncertainties (Data × 100) N = 1684		Radii of the Conoid Surface Fit		Asphericity of the Conoid Surface Fit		Radii of the Biconic Surface Fit		Asphericity of the Biconic Surface Fit	
		CR_x_ in mm	CR_y_ in mm	CQ_x_	CQ_y_	BR_x_ in mm	BR_y_ in mm	BQ_x_	BQ_y_
Corneal front surface	Mean	0.2999	0.3025	0.8317	0.8133	0.2517	0.2389	1.0606	0.9834
	SD	0.9090	0.9131	0.3579	0.3689	0.0967	0.0726	0.4553	0.3362
	Median	0.1798	0.1808	0.7754	0.7585	0.2383	0.2272	0.9995	0.9377
	Quantile 5%	0.1199	0.230	0.5220	0.5135	0.1487	0.1471	0.5236	0.4847
	Quantile 95%	0.4801	0.4879	1.4678	1.4322	0.4291	0.4031	1.8928	1.7336
Corneal back surface	Mean	0.7314	0.7367	0.9631	0.9358	0.3102	0.2941	1.0336	0.9472
	SD	1.9631	1.9518	0.5367	0.6433	0.1107	0.1263	0.4221	0.4422
	Median	0.2216	0.2346	0.8467	0.8046	0.2931	0.2745	0.9582	0.8904
	Quantile 5%	0.1532	0.1673	0.5851	0.5575	0.1884	0.1820	0.5193	0.4634
	Quantile 95%	9.6643	9.2566	2.1749	2.2237	0.5483	0.5108	1.9516	1.7513

**Table 4:** Explorative data of the mean, SD, median, and rms fit error as the difference in height between the measurement data transformed to the canonical coordinates (X_c_/Y_c_/Z_c_) and the surface representation with a conoid (left columns) or a biconic (right columns) for the corneal front and back surface. SD refers to the standard deviation and Quantile 5% and Quantile 95% to the lower and upper bound of the 90% confidence interval.

Fit Error Conoid and Biconic Surface N = 1684		Conoid Surface Fit in µm				Biconic Surface Fit in µm			
		Mean	SD	Median	rms	Mean	SD	Median	rms
Corneal front surface	Mean	−0.0001	1.4322	−0.0348	1.4320	0.0000	1.4023	−0.0339	1.4022
	SD	0.0006	0.4919	0.0812	0.4919	0.0000	0.4801	0.0714	0.4800
	Median	−0.0001	0.3528	−0.0308	1.3527	0.0000	1.3240	−0.0310	1.3238
	Quantile 5%	−0.0003	0.9471	−0.1801	0.9470	0.0000	0.9269	−0.1841	0.9268
	Quantile 95%	0.0000	2.3346	0.0959	2.3344	0.0000	2.2486	0.1018	2.2483
Corneal back surface	Mean	−0.0008	2.7186	0.0023	2.7183	0.0000	2.6307	−0.0093	2.6304
	SD	0.0036	1.1860	0.1794	1.1858	0.0000	1.1377	0.1811	1.1376
	Median	−0.0004	2.4918	0.0059	2.4915	0.0000	2.4205	−0.0060	2.4202
	Quantile 5%	−0.0024	1.8803	−0.2152	1.8801	0.0000	1.8498	−0.2275	1.8496
	Quantile 95%	−0.0002	4.7973	0.2299	4.7968	0.0000	4.4671	0.2304	4.4666

## Data Availability

Data could be provided on serious request from the authors.
